# IL-21 gene rs6822844 polymorphism and rheumatoid arthritis susceptibility

**DOI:** 10.1042/BSR20191449

**Published:** 2020-01-06

**Authors:** Menglei Yu, Jingyi Hou, Minghui Zheng, Yi Cao, Yamuhanmode Alike, Yuanyuan Mi, Jie Zhu

**Affiliations:** 1 Guangdong Provincial Key Laboratory of Malignant Tumor Epigenetics and Gene Regulation, Emergency Department, Sun Yat-Sen Memorial Hospital, Sun Yat-Sen University, Guangzhou, PR China; 2 Guangdong Provincial Key Laboratory of Malignant Tumor Epigenetics and Gene Regulation, Department of Orthopedic, Sun Yat-Sen Memorial Hospital, Sun Yat-Sen University, Guangzhou, PR China; 3 Guangdong Provincial Key Laboratory of Malignant Tumor Epigenetics and Gene Regulation, Department of Clinical Laboratory, Sun Yat-Sen Memorial Hospital, Sun Yat-Sen University, Guangzhou, PR China; 4 Department of Urology, Affiliated Hospital of Jiangnan University, Hefeng Rd, Wuxi 214000, PR China

**Keywords:** anti-citrullinated protein antibody, Interleukin-21, meta-analysis, polymorphism, rheumatoid arthritis, rheumatoid factor

## Abstract

Interleukin-21 (IL-21) is a cytokine that plays a crucial role in pathogenesis and activity of the rheumatoid arthritis (RA). Meanwhile, genetic polymorphisms in the IL-21 gene may alter its expression. Previous studies have reported conflicting results assessing the association between the *IL-21* rs6822844 G/T polymorphism and RA risk. Thus, it’s necessary to perform a meta-analysis to definite above relationship. PubMed database was searched for all papers published until October 20, 2019. Nine case–control studies with 9998 cases and 10742 controls were retrieved based on the search criteria at last. Odds ratio (95% confidence interval) was used to calculate the strength of this association. Publication bias was detected using both Begg’s and Egger’s tests. Overall, the *IL-21 rs6822844* G/T polymorphism was found to be significantly associated with decreased RA risk (e.g. T-allele versus G-allele: OR = 0.81, 95% CI = 0.72–0.91, *P* < 0.001). In addition, decreased RA risk was also detected both in Asians (eg: TT+TG versus GG: OR = 0.42, 95% CI = 0.31–0.56, *P* < 0.001) and Caucasians (eg: TT+TG versus GG: OR = 0.85, 95% CI = 0.80–0.91, *P* < 0.001). A similar trend in association was found in the source of the control and genotype method subgroups. Furthermore, subgroup analysis of rheumatoid factor status revealed a protective relationship between the *IL-21* rs6822844 G/T polymorphism and RF+/RF- RA risk. A similar relationship was noted in the anti-citrullinated protein antibody status subgroup. The results of the present study suggest that the *IL-21* rs6822844 G/T polymorphism was significantly associated with decreased RA susceptibility.

## Introduction

Rheumatoid arthritis (RA) is a relatively common chronic multifactorial autoimmune disorders characterized by progressive damage to joint and tendons, and complications in some case may lead to premature mortality [1]. The etiology of RA remains complex and unknown, but both genetic background and environmental risk factors play a great role in RA risk [2–6]. The genetic contribution to RA is estimated to be between 50% and 60%, and therefore identification of susceptibility genes is important for understanding the biological mechanisms of RA pathogenesis, etiology, prognosis and outcomes [7,8].

Genome wide association studies (GWAS) involving approximately 2000 patients and 3000 controls revealed that the polymorphism *rs6822844 G/T* of the interleukin-21 (IL-21) gene has been defined as a candidate genetic marker with RA risk [9]. The IL-21 gene, also known as Za11 or CVID11, is located on human chromosome 4q27, and encodes a member of the common-γ chain family of cytokines with immunoregulatory activity [10]. The protein encoded by IL-21 is known to be involved in both innate and adaptive immune responses by inducing the differentiation, proliferation and activity of multiple target cells, including macrophages, natural killer cells, B cells and cytotoxic T cells [11–14]. Dysregulation of this gene may result in multiple immune-mediated diseases including RA, systemic lupus erythematosus, psoriasis and chronic inflammatory diseases [15–17].

The *rs6822844* polymorphism locates in the flanking 3′-untranslated region of IL-21 [18], and may regulate the transcription and expression of the IL-21 gene, which may influence the occurrence and progress of RA. Although numbers of studies have evaluated the association between *rs6822844* and RA susceptibility, reported results remain inconsistent [9,19–26]. At the same time, considering the importance of *rs6822844* G/T polymorphism in pathogenesis of RA, it is necessary and urgent to perform a comprehensive meta-analysis of all case–control studies that included both each genotype and eligible factors such as rheumatoid factor (RF) and anti-citrullinated protein antibody (ACPA) status and levels.

## Materials and methods

### Identification of eligible studies

Searches for published data up to October 20, 2019 were conducted on the PubMed (http://www.ncbi.nlm.nih.gov/pubmed) database, without any restriction on language or publication year. The following keywords were used: ‘IL-21 or interleukin-21’, ‘polymorphism or variant’, ‘rheumatoid arthritis or RA’, without any restriction on language or publication year. Using above information, a total of 23 articles were identified. In addition, we also screened references cited in the retrieved articles and other review articles by hand. Studies were selected based on the following inclusion criteria: (1) the study investigated the association between RA and *IL-21 rs6822844* G/T polymorphism; (2) the study was of a case–control design; (3) sufficient genotype numbers (GG, GT and TT) of cases and controls; (4) RF and/or ACPA information was available.

### Data extraction

The following information was collected from eligible publications: the last name of first author, year of publication, country of origin, each genotype number in the case and control group, source of control group, Hardy–Weinberg equilibrium (HWE) of controls, and genotyping methods. In addition, RA diagnostic information, such as autoantibody status (RF+/- or ACPA+/-) was also collected.

### Quality assessment and Newcastle–Ottawa Scale

The quality of the included studies was evaluated by the following five aspects: source of cases, source of controls, specimens used for determining genotypes, total sample size and HWE in controls. The quality scores ranged from 0 to 15, higher scores indicating better quality. Reports scoring <10 were classified as ‘low quality’ and those ≥10 as ‘high quality’ [27]. Besides, the Newcastle–Ottawa Scale (NOS) was also used to assess the quality of each study. This measure assesses observational studies on measures of study quality, such as the selection of cases, comparability of populations and ascertainment of exposure to risks. The NOS ranges from 0 (worst) to 9 stars (best) [28]. Studies with a score of ≥7 stars were considered as high-quality.

### Statistical analysis

Odds ratio (OR) with 95% confidence interval (CI) were used to measure the strength of the association between *IL-21 rs6822844 G/T* polymorphism and RA. The status of RF and ACPA was classified into four categories namely, RF-positive (+) RA, RF-negative (-) RA, ACPA (+) RA and ACPA (-) RA. The statistical significance of the summary OR was determined by using the *Z*-test. A *P* value greater than 0.10 for the *Q*-test indicated a lack of heterogeneity among the studies. In cases where significant heterogeneity was detected, the random effects model was used, otherwise the fixed effects model was used [29,30]. We investigated the association between IL-21 variant and RA risk: the allelic contrast (T-allele versus G-allele), homozygote comparison (TT versus GG), dominant genetic model (TT+TG versus GG), heterozygote comparison (TG versus GG) and recessive genetic model (TT versus TG+GG). Funnel plot asymmetry was assessed using Begg’s test and publication bias was assessed using Egger’s test. In both tests *P* < 0.05 was considered statistically significant [31]. HWE was evaluated using Pearson’s chi-square (*χ*^2^) test, where *P* < 0.05 was considered significant [32]. All statistical tests used in the present study were performed using Stata (version 11.0; StataCorp LP, College Station, TX). The power and sample size analysis of our meta-analysis was calculated by a program called PS: Power and Sample Size Calculation (http://biostat.mc.vanderbilt.edu/wiki/Main/PowerSampleSize#Windows) [33].

## Results

### Characteristics of selected studies

**Figure 1:**
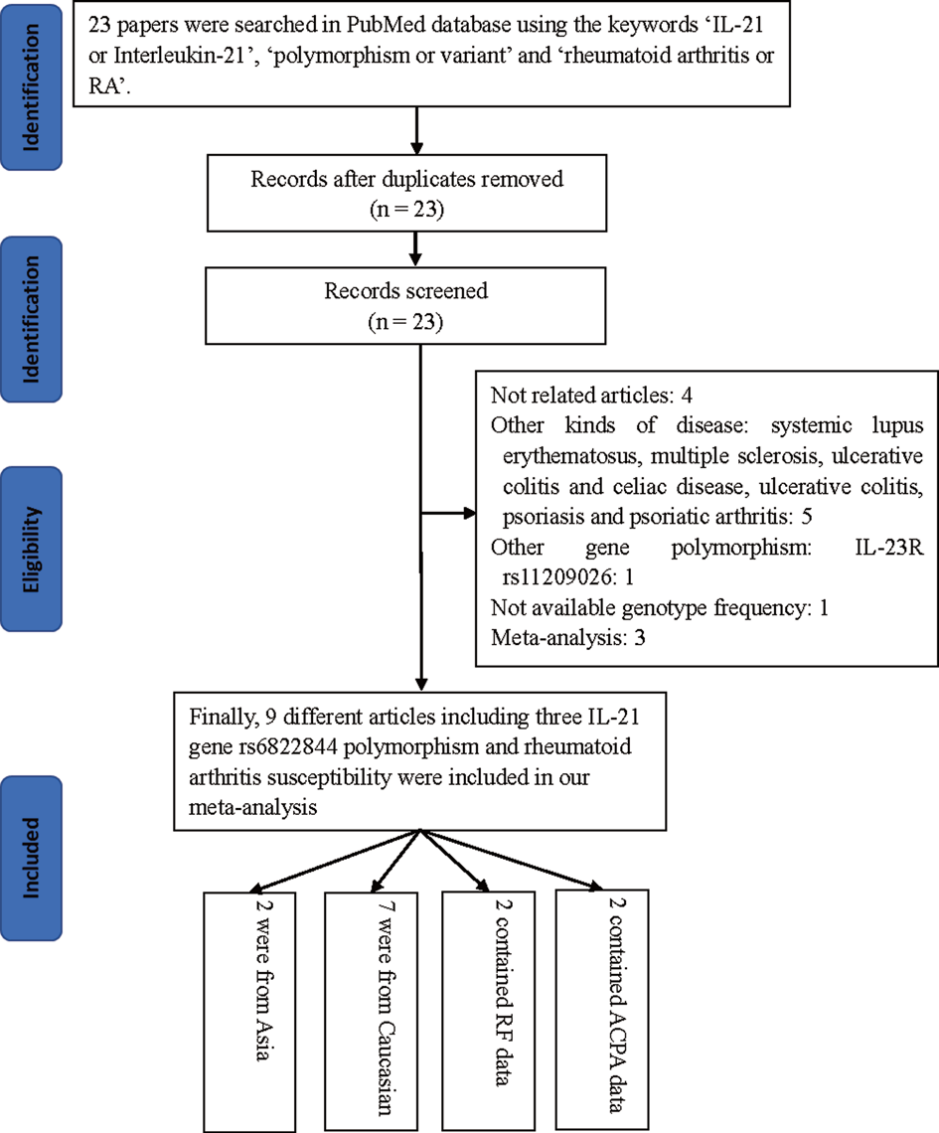
A flowchart illustrating the search strategy used to identify association studies for *IL-21 rs6822844* polymorphism and RA risk

**Figure 2:**
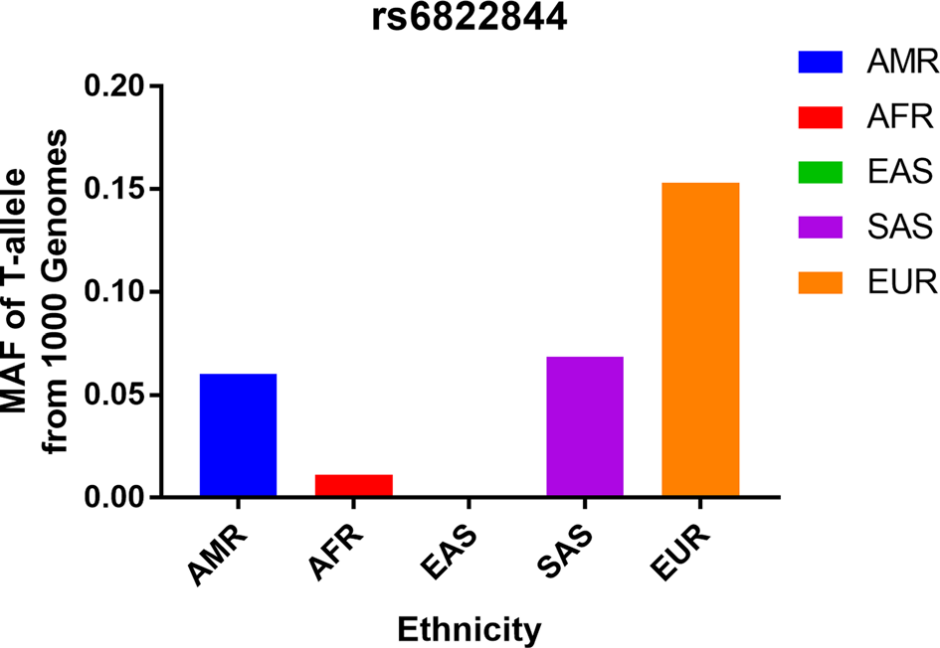
T-allele frequencies for the *IL-21 rs6822844* polymorphism among cases/controls stratified by ethnicity Vertical line, T-allele frequency; Horizontal line, ethnicity type. EAS: East Asian; EUR: European; AFR: African; AMR: American; SAS: South Asian.

A total of 23 published articles were retrieved from the PubMed database in accordance with our selection criteria. Among those, 11 articles were excluded for the following reasons: four articles were unrelated; five articles were excluded because they investigated diseases other than RA; one examined the *IL-23R rs11209026* polymorphism, and one did not have complete genotype’s information, three articles were classified to meta-analysis. Following the above screening, the remaining nine articles, comprising a total of 9998 cases and 10,742 controls, were selected for the meta-analysis (Table 1 and Figure 1) [9,19–26]. Seven studies evaluated Caucasian subjects and the remaining two investigated Asian subjects. In addition, two of the studies included information about RF and ACPA status [20,22]. All of the patients studied here were diagnosed according to the American College of Rheumatology’s revised criteria [34]. The controls were unrelated, healthy individuals, who were age and ethnically matched. Polymorphisms were genotyped using TaqMan genotyping assays. Characteristics of *IL-21 rs6822844* G/T polymorphism studies are summarized in Tables 1 and 2. The genotype frequencies in the controls were in Hardy–Weinberg equilibrium, except in one study [20]. Finally, we confirmed that our analysis is of adequate statistical power (0.998). Finally, we checked the Minor Allele Frequency (MAF) reported for the five main worldwide populations in the 1000 Genomes Browser (https://www.ncbi.nlm.nih.gov/snp/rs6822844#frequency_tab): East Asian (EAS), 0.001; European (EUR), 0.1531; African (AFR), 0.0113; American (AMR), 0.0605; and South Asian (SAS), 0.0685 (Figure 2). The MAF in our analysis was 0.1542 and 0.1790 in the case and control groups, respectively, both higher than the results from1000 Genomes Browser database.

**Table 1:** Study characteristics from published studies on the relationship between *IL-21 rs6822844* polymorphism and RA risk

Author	Year	Country	Ethnicity	Case	Control	SOC	Case			Control			HWE	Genotype	Quality Score	NOS
							TT	TG	GG	TT	TG	GG				
Daha	2009	Netherlands	Caucasian	877	866	HB	116	53	708	126	73	667	<0.01	MALDI-TOF-MS	6	6
Zhernakova	2007	Netherlands	Caucasian	1012	924	PB	21	243	748	31	280	613	0.984	SNaPshot	8	8
WTCCC	2007	U.K.	Caucasian	1856	2933	PB	61	553	1242	94	958	1881	0.035	GeneChip	10	7
Maiti	2010	Turkey	Asian	354	368	HB	6	32	316	4	65	299	0.824	TaqMan	11	7
Barton	2009	U.K.	Caucasian	3886	3454	HB	95	1052	2739	125	1003	2326	0.193	TaqMan	10	7
Louahchi	2016	Algeria	Asian	323	323	PB	6	31	286	10	80	233	0.336	TaqMan	10	9
Malinowski	2017	Poland	Caucasian	422	338	PB	6	103	313	4	79	255	0.438	TaqMan	10	9
Teixeira	2009	France	Caucasian	434	434	PB	8	99	327	11	110	313	0.719	TaqMan	10	8
Hollis-Moffatt	2010	New Zealand	Caucasian	834	1102	PB	30	221	583	29	330	743	0.285	TaqMan	11	8

Abbreviations: HB, hospital-based; HWE, Hardy–Weinberg equilibrium of control group; MALDI-TOF MS, polymerase chain reaction–matrix-assisted laser desorption/ionization time-of-flight mass spectrometry; NOS, Newcastle–Ottawa Scale; PB, population-based; SOC, source of control.

**Table 2:** RA characteristics from published studies on the relationship for *IL-21 rs6822844* polymorphism

Author	Year	Ethnicity	Types	Case	Control	Case			Control		
						TT	TG	GG	TT	TG	GG
Louahchi	2016	African	ACPA^−^	73	323	9	9	55	10	80	233
Daha	2009	Caucasian	ACPA^−^	228	866	8	60	160	126	73	667
Louahchi	2016	African	ACPA^+^	258	323	6	26	226	10	80	233
Daha	2009	Caucasian	ACPA^+^	327	866	25	52	250	126	73	667
Daha	2009	Caucasian	RF^−^	250	866	20	38	192	126	73	667
Louahchi	2016	African	RF^−^	101	323	1	7	93	10	80	233
Daha	2009	Caucasian	RF^+^	487	866	55	53	379	126	73	667
Louahchi	2016	African	RF^+^	222	323	5	23	194	10	80	233

Abbreviations: ACPA, anti-citrullinated protein antibody; RF, rheumatoid factor.

### Pooled analyses

**Figure 3:**
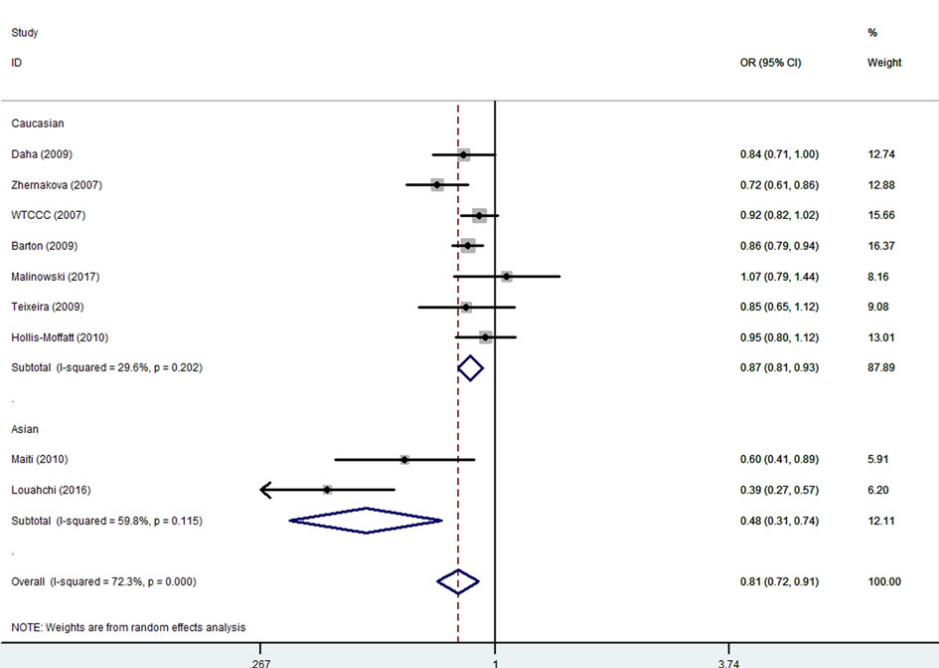
Forest plot of RA risk associated with *IL-21 rs6822844* polymorphism (T-allele versus G-allele) in the overall analysis The squares and horizontal lines correspond to the study-specific OR and 95% CI. The area of the squares reflects the weight (inverse of the variance). The diamond represents the summary OR and 95% CI.

Overall, a significant protective association was observed between the variant genotypes of *IL-21 rs6822844* G/T and RA risk in all different genetic models. In the allelic contrast model [OR = 0.81, 95% CI = 0.72–0,91, *P* value of heterogeneity test (*P*_h_) < 0.001, *P* value of *z*-test <0.001] and complete dominant model [OR = 0.78, 95% CI = 0.68–0.89, *P* value of heterogeneity test (*P*_h_) < 0.001, *P* value of *z*-test < 0.001] (Figure 3), and the dominant model [OR = 0.96, 95% CI = 0.95–0.97, *P*_h_ = 0.123] (Table 3).

**Table 3:** Total and stratified analysis of *IL-21 rs6822844* polymorphism and RA risk

Variables	*N*	Case/Control	T-allele versus G-allele			TG versus GG			TT versus GG			TT+TG versus GG			TT versus TG+GG		
			OR (95%CI)	*P*_h_	*P*	OR (95%CI)	*P*_h_	*P*	OR (95%CI)	*P*_h_	*P*	OR (95%CI)	*P*_h_	*P*	OR (95%CI)	*P*_h_	*P*
Total	9	9998/10742	0.81 (0.72–0.91)	<0.001	<0.001	0.76 (0.65–0.88)	<0.001	<0.001	0.81 (0.70–0.94)	0.176	0.005	0.78 (0.68–0.89)	<0.001	<0.001	0.85 (0.73–0.98)	0.205	0.027
Ethnicity																	
Asian	2	677/691	0.48 (0.37–0.63)	0.115	<0.001	0.38 (0.28–0.53)	0.231	<0.001	0.74 (0.34–1.62)	0.202	0.456	0.42 (0.31–0.56)	0.148	<0.001	0.87 (0.40–1.89)	0.243	0.721
Caucasian	7	9321/10051	0.87 (0.83–0.92)	0.202	<0.001	0.86 (0.80–0.92)	0.331	<0.001	0.81 (0.70–0.95)	0.134	0.005	0.85 (0.80–0.91)	0.335	<0.001	0.85 (0.73–0.98)	0.143	0.029
SOC																	
HB	3	5117/4688	0.84 (0.78–0.91)	0.218	<0.001	0.69 (0.48–1.00)	0.011	0.050	0.76 (0.63–0.92)	0.202	0.004	0.77 (0.63–0.95)	0.073	0.017	0.78 (0.65–0.95)	0.181	0.012
PB	6	4881/6054	0.81 (0.67–0.97)	<0.001	0.023	0.77 (0.62–0.94)	<0.001	0.012	0.90 (0.71–1.13)	0.219	0.355	0.77 (0.63–0.95)	<0.001	0.014	0.95 (0.76–1.19)	0.317	0.665
Genotype methods																	
Others	3	3745/4723	0.83 (0.72–0.96)	0.067	0.010	0.81 (0.73–0.90)	0.151	<0.001	0.86 (0.70–1.04)	0.229	0.128	0.82 (0.75–0.90)	0.123	<0.001	0.90 (0.74–1.09)	0.293	0.273
Taqman	6	6253/6019	0.78 (0.64–0.95)	<0.001	0.015	0.73 (0.56–0.93)	<0.001	0.012	0.76 (0.61–0.95)	0.164	0.014	0.74 (0.59–0.94)	<0.001	0.012	0.79 (0.64–0.99)	0.167	0.037
RF status																	
RF+	2	709/1189	0.63 (0.32–1.24)	0.004	0.181	0.67 (0.19–2.43)	<0.001	0.544	0.75 (0.54–1.04)	0.673	0.085	0.61 (0.24–1.53)	0.001	0.292	0.75 (0.54–1.03)	0.950	0.074
RF-	2	351/1189	0.47 (0.15–1.46)	0.003	0.194	0.65 (0.08–5.44)	<0.001	0.689	0.52 (0.32–0.84)	0.466	0.008	0.49 (0.11–2.22)	<0.001	0.357	0.49 (0.31–0.80)	0.651	0.004
ACPA status																	
ACPA+	2	585/1189	0.60 (0.33–1.09)	0.010	0.096	0.80 (0.15–4.43)	<0.001	0.801	0.54 (0.36–0.82)	0.786	0.004	0.62 (0.23–1.72)	<0.001	0.362	0.52 (0.34–0.78)	0.454	0.002
ACPA-	2	301/1189	0.94 (0.75–1.20)	0.196	0.635	1.31 (0.18–9.33)	<0.001	0.786	0.99 (0.07–14.35)	<0.001	0.993	1.25 (0.94–1.66)	0.128	0.121	0.96 (0.05–20.27)	<0.001	0.977

*P*_h_: value of *Q*-test for heterogeneity test; *P*: *Z*-test for the statistical significance of the OR; ACPA: anti-citrullinated protein antibody;RF:rheumatoid factor; HB: hospital-based; PB: population-based; SOC; source of control

**Figure 4:**
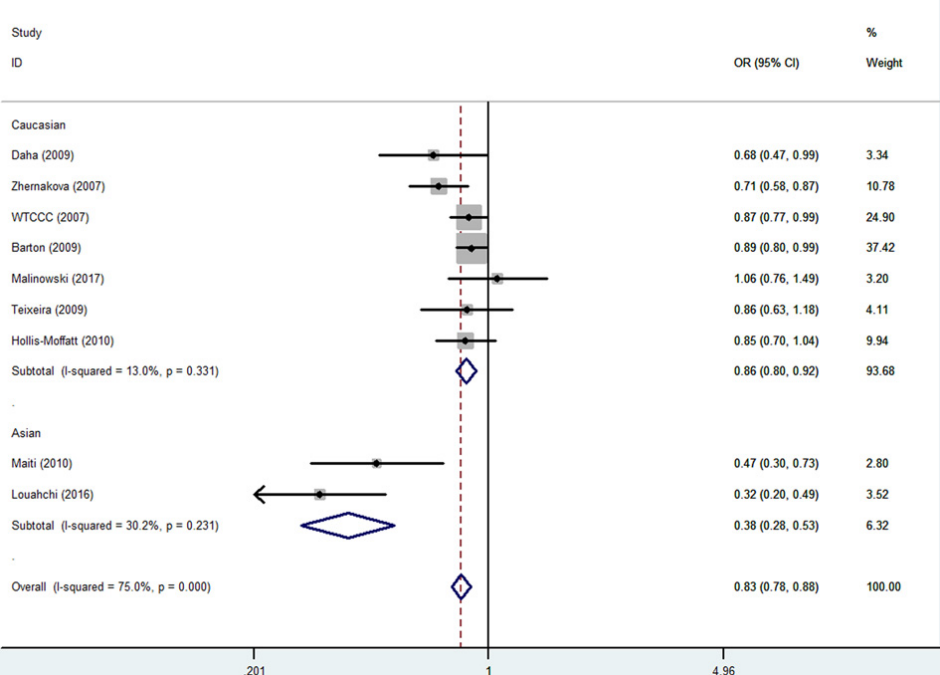
Forest plot of RA risk associated with *IL-21 rs6822844* polymorphism (TG versus GG) in the ethnicity subgroup The squares and horizontal lines correspond to the study-specific OR and 95% CI. The area of the squares reflects the weight (inverse of the variance). The diamond represents the summary OR and 95% CI.

In the stratified analysis by ethnicity, a decreased association was observed both in Asian [e.g. TG versus GG: OR = 0.38, 95% CI = 0.28–0.53, *P* value of heterogeneity test (*P*_h_) = 0.231, *P* value of *z*-test <0.001] and Caucasian populations [TG versus GG: OR = 0.86, 95% CI = 0.80–0.92, *P* value of heterogeneity test (*P*_h_) = 0.331, *P* value of *z*-test <0.001] (Figure 4) (Table 3).

**Figure 5:**
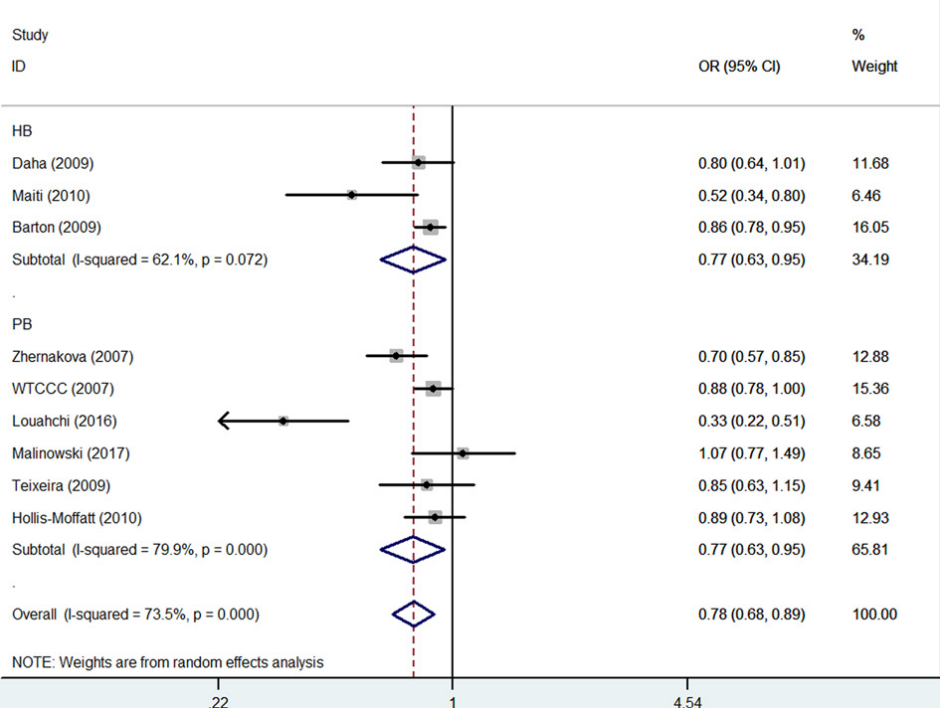
Forest plot of RA risk associated with *IL-21 rs6822844* polymorphism (TT+TG versus GG) in the source of control The squares and horizontal lines correspond to the study-specific OR and 95% CI. The area of the squares reflects the weight (inverse of the variance). The diamond represents the summary OR and 95% CI.

In addition, in the stratified analysis by the source of the control, similar results were detected in both HB [TT+TG versus GG: OR = 0.77, 95% CI = 0.63–0.95, *P* value of heterogeneity test (*P*_h_) = 0.073, *P* value of *z*-test = 0.017] and PB [TT+TG versus GG: OR = 0.77, 95% CI = 0.63–0.95, *P* value of heterogeneity test (*P*_h_) < 0.001, *P* value of *z*-test = 0.014] (Figure 5) (Table 3).

**Figure 6:**
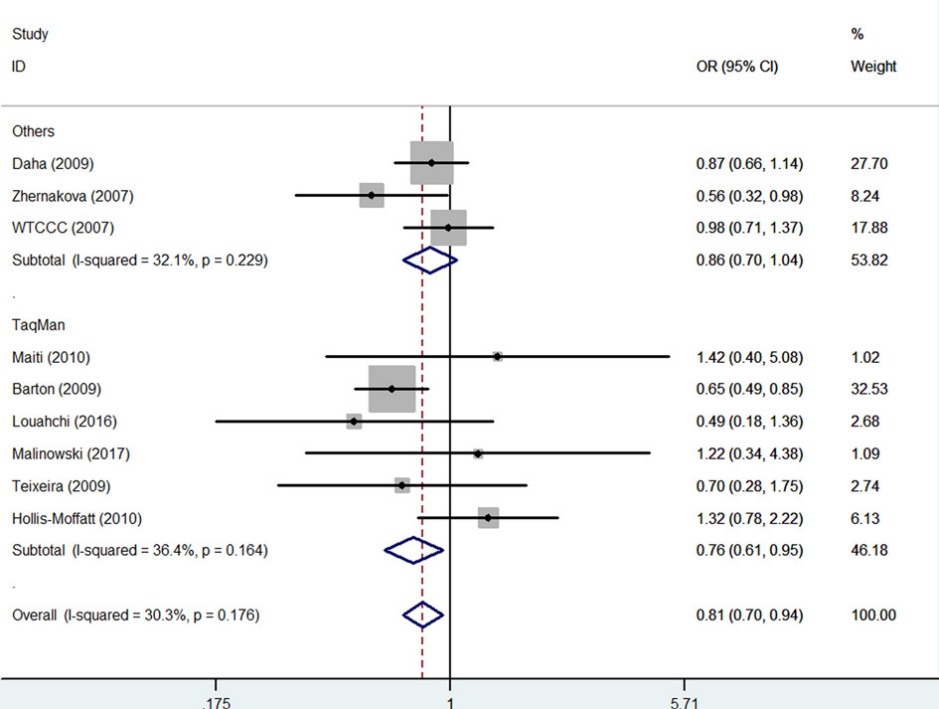
Forest plot of RA risk associated with *IL-21 rs6822844* polymorphism (TT versus GG) in the genotype methods subgroup The squares and horizontal lines correspond to the study-specific OR and 95% CI. The area of the squares reflects the weight (inverse of the variance). The diamond represents the summary OR and 95% CI.

Several different methods were applied in the included studies. We therefore analyzed whether association was existed in variant methods, significant association was observed using TaqMan [TT versus GG: OR = 0.76, 95% CI = 0.61–0.95, *P* value of heterogeneity test (*P*_h_) = 0.164, *P* value of *z*-test = 0.014] (Figure 6) (Table 3)

**Figure 7:**
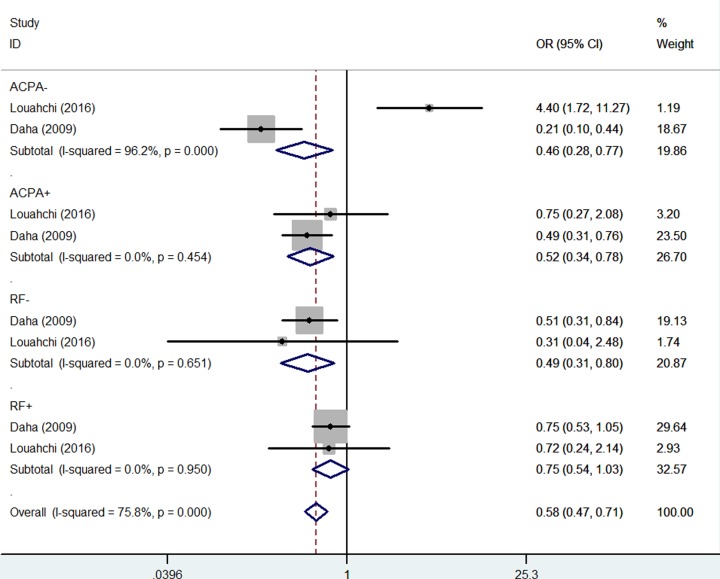
Forest plot of RA risk associated with *IL-21 rs6822844* polymorphism (TT versus TG+GG) in the autoantibody subgroup (RF and ACPA status) The squares and horizontal lines correspond to the study-specific OR and 95% CI. The area of the squares reflects the weight (inverse of the variance). The diamond represents the summary OR and 95% CI.

Finally, in the stratified analysis by RF status, pooled associations were found among RF^−^ RA risk and *IL-21 rs6822844 G/T* polymorphism [OR = 0.52, 95% CI = 0.32–0.84, *P*_h_ = 0.466, *P* = 0.008 for TT versus GG, and OR = 0.49, 95% CI = 0.31–0.80, *P*_h_ = 0.651, *P* = 0.004 for TT versus GG, Figure 7]. Similarly, significant associations were also detected in several models in ACPA+ status [OR = 0.52, 95% CI = 0.34–0.78, *P*_h_ = 0.454, *P* = 0.002 for recessive model, Figure 7] (Table 3).

### Publication bias and sensitivity analysis

**Figure 8:**
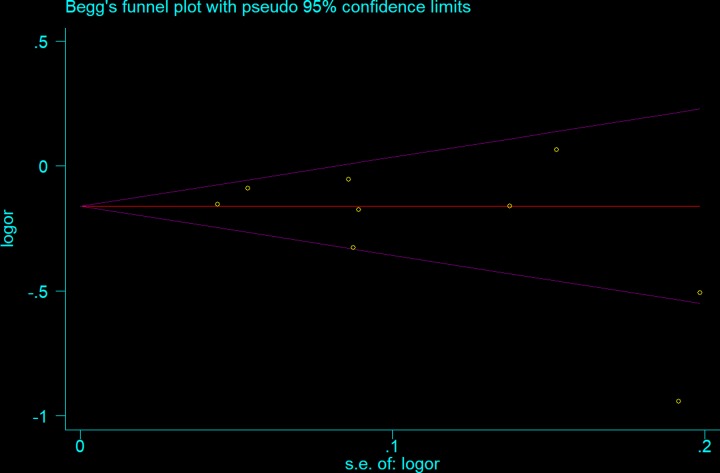
Begg’s funnel plot for publication bias test (T-allele versus G-allele)

**Figure 9:**
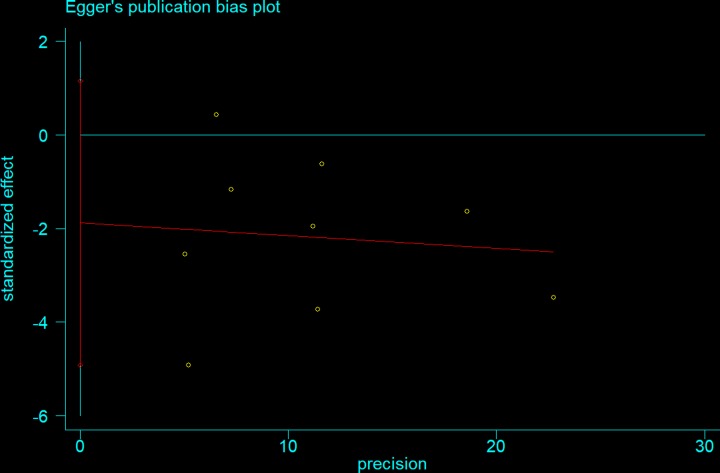
Egger’s publication bias plot (T-allele versus G-allele)

**Figure 10:**
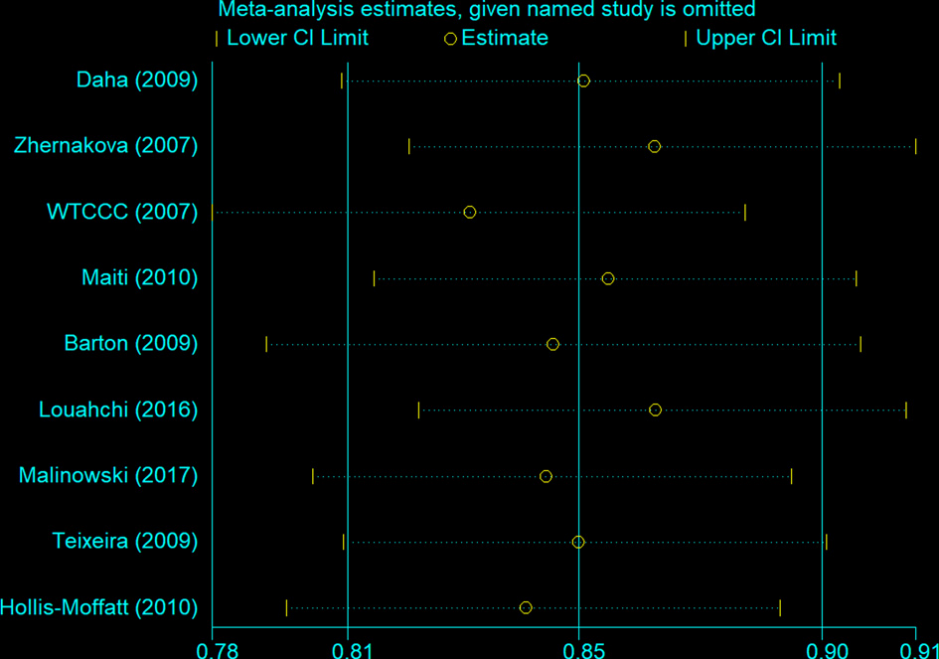
Sensitivity analysis between *IL-21 rs6822844* polymorphism and RA risk (T-allele versus G-allele)

Begg’s and Egger’s tests were performed to assess publication bias. As shown in Table 4, neither of the tests provided any evidence of publication bias (T-allele versus G-allele: *t* = −1.47, *P* = 0.186 for Egger’s test; and *z* = 1.15, *P* = 0.251 for Begg’s test, Figures 8 and 9). Sensitivity analysis was performed to assess whether individual studies influenced the pooled ORs by sequential removal of individual studies. The results suggested that no single study significantly affected the overall OR (Figure 10).

**Table 4:** Publication bias tests (Begg’s funnel plot and Egger’s test for publication bias test)

Egger’s test						Begg’s test	
Genetic type	Coefficient	Standard error	*t*	*P-*value	95%CI of intercept	*z*	*P-*value
T-allele versus G-allele	−1.883	1.283	−1.47	0.186	(−4.918,1.152)	1.15	0.251
TG versus GG	−1.427	1.049	−1.36	0.261	(−3.906,1.053)	1.36	0.175
TT versus GG	−0.534	0.413	−1.29	0.237	(−1.511,0.443)	0.73	0.466
TT+TG versus GG	−1.671	1.157	−1.44	0.192	(−4.407,1.063)	0.94	0.348
TT versus TG+GG	−0.535	0.414	−1.29	0.237	(−1.514,0.443)	0.94	0.348

for *IL-21 rs6822844* polymorphism.

## Discussion

To our knowledge, RA is a systemic, inflammatory autoimmune disorder with numerous symptoms caused by an intricate chain of physiological events [35]. Tumor necrosis factor α and interleukin family (such as interleukin 1β, interleukin 17 and interleukin 21) are some of the key mediators of RA pathogenesis [36]. In recent years, *IL-21* has been found to be a key player in RA pathogenesis and progression [37–39]. In RA pathogenesis, IL-21 receptor (IL-21R) is highly expressed in CD4+ T cell subsets, macrophages, dendritic cells and synovial fibroblasts [40]. These immune cell subtypes recognize the IL-21 in the microenvironment and carry out several intricate chains of events [41]. IL-21 has been implicated to be an important target in RA therapy, and several studies have substantiated its role through activation of signaling pathways and in promoting inflammatory condition [42,43].

The Wellcome Trust Case Control Consortium (WTCCC) first designed and analyzed GWAS studies, comprising 2000 cases and 3000 shared controls, and found a significant association between a common haplotype located in the 3′-untranslated region of *IL-21* and susceptibility to RA [9]. Following this, several studies re-analyzed this association using a greater number of samples to increase statistical power, in addition to including other predictors, such as RF and ACPA status and levels. However, results have been controversial. Zhernakova et al. [26] detected a decrease in frequency of the *rs6822844* T-allele in RA (14.1%), and demonstrated significant association between this polymorphism and RA susceptibility (OR = 0.72, 95% CI = 0.61–0.86, *P* < 0.001). Moreover, Louahchi et al. [22] showed a protective effect of the minor T-allele (OR = 0.39, 95% CI = 0.26–0.57), whereas the major G-allele appeared to be a risk susceptibility (OR = 2.57, 95% CI = 1.74–3.83, *P* < 0.001). Nevertheless, Malinowski et al. [24] examined 422 RA cases and 338 controls, and found that *IL-21 rs6822844* was not risk loci for RA susceptibility.

To our knowledge, the present study was combined to investigate whether there has an association between *IL-21 rs6822844* polymorphism and RA risk. We performed a meta-analysis involving 9998 RA cases and 10742 controls. The main finding of our study was that *rs6822844* T-allele was a protective factor for individuals carrying this allele, in other words, the *rs6822844* polymorphism in Asian and Caucasian populations may decrease RA susceptibility in these ethnic groups. If an individual carries the IL-21 *rs6822844* T-allele, he/she may have a lower risk to become RA patient than G-allele, in contrast, G-allele carries may have a higher risk to become RA. Through detected this polymorphism, we may know the susceptibility of RA for one person in advance, which may be helpful or make sense in the future. Our results suggest that the *rs6822844* polymorphism influenced not only the RF status, but also the presence of ACPA. Determination of any genetic differences between RF and ACPA status in RA patients may shed light on their roles in RA pathogenesis. Such data may be useful in the clinical diagnosis and prevention of RA, as well as for identifying therapeutic targets in the treatment of RA patients. Recently, Mohammadi et al. also made Bayesian meta-analysis about polymorphisms of inflammatory cytokines and RA risk; however, positive results were found about genetic polymorphisms of IL23R gene, but not IL-21 and IL17A genes. Moreover, RF and ACPA status were not involved [44], which were the different and innovate about present study.

Although the results reported here were statistically significant, there are some limitations in our study should be mentioned. First, the number of publications included remains insufficient for a comprehensive analysis. Second, gene–gene and gene–environment interactions were not taken into consideration here. In addition, other covariates including age, sex, family history, environmental factors, disease stage and lifestyle also should be included. Third, the distribution of the ethnicity and source of control categories was not balanced, especially in Asian populations and HB. Fourth, the number of studies reporting RF and ACPA status was not very large. Fifth, following items: the correlation of *IL-21 rs6822844* polymorphism with the stages of arthritis; the protein expression level of IL-21 in different polymorphisms should also be added and be re-analyzed by meta in the future research. Sixth, IL-12 gene contains many SNPs, in our included nine studies, three studies contained two or more SNPs, for example Malinowski et al., (2016) contained *rs6822844, rs2221903, rs6840978* and *rs2285452*; Teixeira et al., (2009) contained not only *rs6822844*, but also *rs4505848, rs11732095, rs4492018*, and *rs1398553*; Hollis-Moffatt et al., (2010) contained only *rs6822844* and *rs907715*. Thus, we can’t analyze the existed interaction between the SNPs in the same IL-21 gene. Despite these limitations, some advantages should also be mentioned. First, all selected studies were published after 2008, which may be indicative of the increasing attention that this topic has garnered in recent years. Second, the coverage of RA features in this meta-analysis was more extensive. Third, our study combined the highest number of individuals so far, the power of our meta-analysis was greater than 0.95, which suggested that the data were trustworthy.

In summary, our study presented evidence that *IL-21 rs6822844* polymorphism was associated with significantly decreased RA risk and that the RF autoantibody may be considered as a biomarker for the detection of RA susceptibility.

## Abbreviations

HWE: Hardy–Weinberg equilibrium
IL-21: interleukin-21
NOS: Newcastle–Ottawa Scale
RA: rheumatoid arthritis
